# The predictive value of p53, p53R2, and p21 for the effect of chemoradiation therapy on oesophageal squamous cell carcinoma

**DOI:** 10.1038/sj.bjc.6602322

**Published:** 2005-01-18

**Authors:** H Okumura, S Natsugoe, M Matsumoto, Y Mataki, H Takatori, S Ishigami, S Takao, T Aikou

**Affiliations:** 1 Department of Surgical Oncology, Digestive Surgery, Graduate School of Medicine, Kagoshima University, Sakuragaoka 8-35-1, Kagoshima 890-8520, Japan

**Keywords:** p53, p53R2, p21, chemoradiation, oesophageal cancer

## Abstract

The p53 family regulates cell-cycle arrest, triggers apoptosis or is involved in repair of DNA damage. In the present study, we analysed the expression of some p53 family proteins and their responses to chemoradiation therapy (CRT) in cases of oesophageal squamous cell carcinoma (ESCC). We immunohistochemically investigated the relationship between p53, p53R2, and p21 expression in biopsy specimens of untreated primary tumours and their clinical and histological responses to CRT in 62 patients with ESCC. Chemoradiation therapy consisted of 5-fluorouracil plus cisplatin and 40 Gy of radiation. The rates of clinical and histological responses (complete or partial) to CRT were 71.0% (clinical) and 52.8% (histological). The rate of positive expression was 43.5% for p53, 37.1% for p53R2, and 54.8% for p21 expression. Statistically significant correlations were found between p53 or p53R2 expression and favourable response to CRT (*P*=0.0001 or 0.041 clinical, *P*=0.016 or 0.0018 histological, respectively). Furthermore, in p53-negative tumours, CRT was more effective in tumours with p53R2 negative expression than those with p53R2 positive expression (*P*=0.0014). We demonstrated that the negative expression of p53 and p53R2 expression was closely related to the effect of CRT and should predict the CRT outcome in patients with ESCC.

Since the prognosis of patients with oesophageal squamous cell carcinoma (ESCC) is still poor, various types of aggressive therapy such as extended lymphadenectomy, radiotherapy, and chemotherapy are being used (Naunheim *et al*, 1992; Baba *et al*, 1994). Chemoradiation therapy (CRT) for the treatment of oesophageal cancer has been investigated since the 1980s, and the combination of 5-fluorouracil (5-FU) and cisplatin has been regarded as an enhancer of radiosensitivity (John *et al*, 1989). Chemoradiation therapy is one of the most useful treatments for ESCC (Nakano *et al*, 2001). As some patients have responded well to CRT and others do not, it is important to predict the CRT response from markers before beginning treatment.

From recent advances in fundamental research, many biological markers concerning apoptosis, DNA repair, and the cell cycle have been elucidated for their association with responses to CRT in cases of ESCC. The effectiveness of CRT may be closely associated with apoptosis (Thompson, 1995). The tumour suppressor gene p53 regulates cell-cycle arrest and triggers apoptosis following DNA damage (Lowe *et al*, 1994). Additionally, p53 is involved in the repair of DNA damage caused by various genotoxic stresses and protects cells from death after irradiation (Caelles *et al*, 1994). The p21 gene is a well-known mediator in the p53 signalling pathway that induces G1 arrest, allowing time for damaged DNA to be repaired (Xiong *et al*, 1993). A recently identified ribonucleotide reductase, p53R2, is directly regulated by p53 for supplying nucleotides to repair damaged DNA. The DNA synthesis in cells arrested in G1 or G2 after DNA damage is mediated by p53R2 (Tanaka *et al*, 2000; Yamaguchi *et al*, 2001).

The aims of this retrospective study were to examine the expression of p53, p53R2, and p21 in biopsy specimens of ESCC and to evaluate whether such expression is useful for predicting the response to CRT.

## MATERIALS AND METHODS

### Study groups

The present study involved 62 consecutive patients with advanced ESCC who underwent CRT at the First Department of Surgery of Kagoshima University Hospital between January 1995 and December 2001. Of these patients, 36 underwent CRT followed by oesophagectomy with lymph node dissection 4–6 weeks after completing CRT, and 26 received only CRT. After all patients gave informed consent, biopsy specimens of the primary tumours were endoscopically collected. Classifications of the specimens were determined according to the International Union against Cancer tumour-node-metastasis (TNM) classification system (Sobin and Wittwkind, 1997). All patients were followed up after discharge with a radiographic examination every 1–3 months, computed tomography every 3–6 months, and ultrasonography every 6 months. Follow-up data after surgery were available for all patients with a median follow-up period of 20 months (range 1–70 months). The clinicopathologic features of the study group are summarised in Table 1. All of the M1 tumours were due to distant lymphnode metastases.

### Chemoradiation therapy

A total radiation dose of 40 Gy was applied; 2 Gy fractions were delivered 5 days per week for 4 weeks to the mediastinum and neck. In the same period, chemotherapy was performed intravenously using two anticancer agents: cisplatin (7 mg over 2 h) and 5-FU (350 mg over 24 h).

The clinical response to CRT was evaluated by the findings of oesophagography, oesophagoscopy, endoscopic ultrasonography, and computed tomography. The clinical criteria for the response were as follows (Japanese Society for Esophageal Diseases, 1999): complete response (CR), disappearance of tumour and continuous effect for more than 4 weeks; partial response (PR), response rate more than 50% and no new lesions for more than 4 weeks; no change (NC), response rate less than 50% or progressive disease (PD) less than 25% and no new lesions for 4 weeks; and PD, progressive disease more than 25% or appearance of new lesions. The patients whose clinical effect was CR or PR were judged as positively susceptible to CRT, whereas the patients with NC or PD were judged as not susceptible.

The histological criteria for the response of CRT were as follows (Japanese Society for Esophageal Diseases, 1999). Grade 0: Neither necrosis nor cellular or structural changes can be seen throughout the lesion. Grade 1: Necrosis or disappearance of the tumour is present in no more than 2/3 of the whole lesion. Grade 2: Necrosis or disappearance of the tumour is present in more than 2/3 of the whole lesion, but viable tumour cells are still remaining. Grade 3: The whole lesion falls into necrosis and/or is replaced by fibrosis, with or without granulomatous changes. No viable tumour cells are observed. In patients whose histological response was Grade 2 or 3, the CRT was judged to be effective. On the other hand, in patients whose histological response was Grade 0 or 1, the CRT was judged to be ineffective.

### Immunohistochemistry

Tumour samples were fixed with 10% formaldehyde in phosphate-buffered saline (PBS), embedded in paraffin, and sectioned into 4-mm thick slices. After deparaffinisation of the sections, the endogenous peroxidase was blocked by immersing the slides in a 0.3% hydrogen peroxidase–methanol solution for 30 min at room temperature. For staining with p53, p53R2, and p21 antibodies, sections were pretreated with citrate buffer for 10 min at 100°C in a microwave oven. The sections were washed with PBS for 5 min three times, and then blocked by treatment with PBS containing 3% skim milk for 30 min at room temperature. The blocked sections were incubated with the diluted primary antibody: p53 (DO7, Novocastra Laboratories, Newcastle upon Tyne, UK), 1 : 50; p53R2 (sc-10840, Santa Cruz Biotechnology, Inc., Santa Cruz, CA, USA), 1 : 100; p21 (sc-817, Santa Cruz Biotechnology, Inc., Santa Cruz, CA, USA), 1 : 100 with PBS at 4°C overnight, followed by staining with a streptavidin–biotin–peroxidase kit (Nichirei, Tokyo, Japan). The sections were washed in PBS for 5 min three times, and the immune complex was visualised by incubating the sections with diaminobenzidine tetrahydrochloride. They were rinsed briefly in water, counterstained with haematoxylin, and mounted.

Evaluation of immunohistochemistry was independently carried out by two investigators (HO and SN). Regarding the immunohistochemical evaluation of p53 or p21, a distinct nuclear immunoreaction in more than 10% of the cancer cells was judged as p53 positive (p53 (+)) or p21 positive (p21 (+)) and in less than 10% of the cancer cells as p53 negative (p53 (−)) or p21 negative (p21 (−)), according to our previously report (Natsugoe *et al*, 1999). For p53R2, cytoplasmic immunoreaction in more than 10% of the cancer cells was judged as p53R2 positive (p53R2 (+)) and in less than 10% of the cancer cells as p53R2 negative (p53R2 (−)).

In ESCC, immunohistochemically detectable p53 protein is frequent due to mutation in the p53 gene, which results in the formation of an abnormal protein with a prolonged half-life. Therefore, p53 (+) immunoreactivity is considered to be the product of a mutated gene for p53, and p53 (−) immunoreactivity is associated with the wild-type p53 gene. (Wagata *et al*, 1993). On the other hand, the p53R2 (+) immunoreactivity indicates basal p53R2 expression and p53R2 (−) immunoreactivity is associated to inactivation of p53R2 that is caused by intragenic mutation (Byun *et al*, 2002).

### Statistical analysis

Associations between two parameters were analysed with the Spearman's rank correlation test. A value of *P*<0.05 was considered to be significant. Actuarial survival curves were estimated using the Kaplan–Meier method, and differences in survival between subgroups were compared with the log-rank test. Multivariate analysis was made using Cox-hazard model analysis. All statistical analyses were performed using the software package StatView™ version 5.0 (Abacus Concepts, Berkeley, CA, USA).

## RESULTS

### Expression of p53, p53R2, and p21 in ESCC

**Figure 1:**
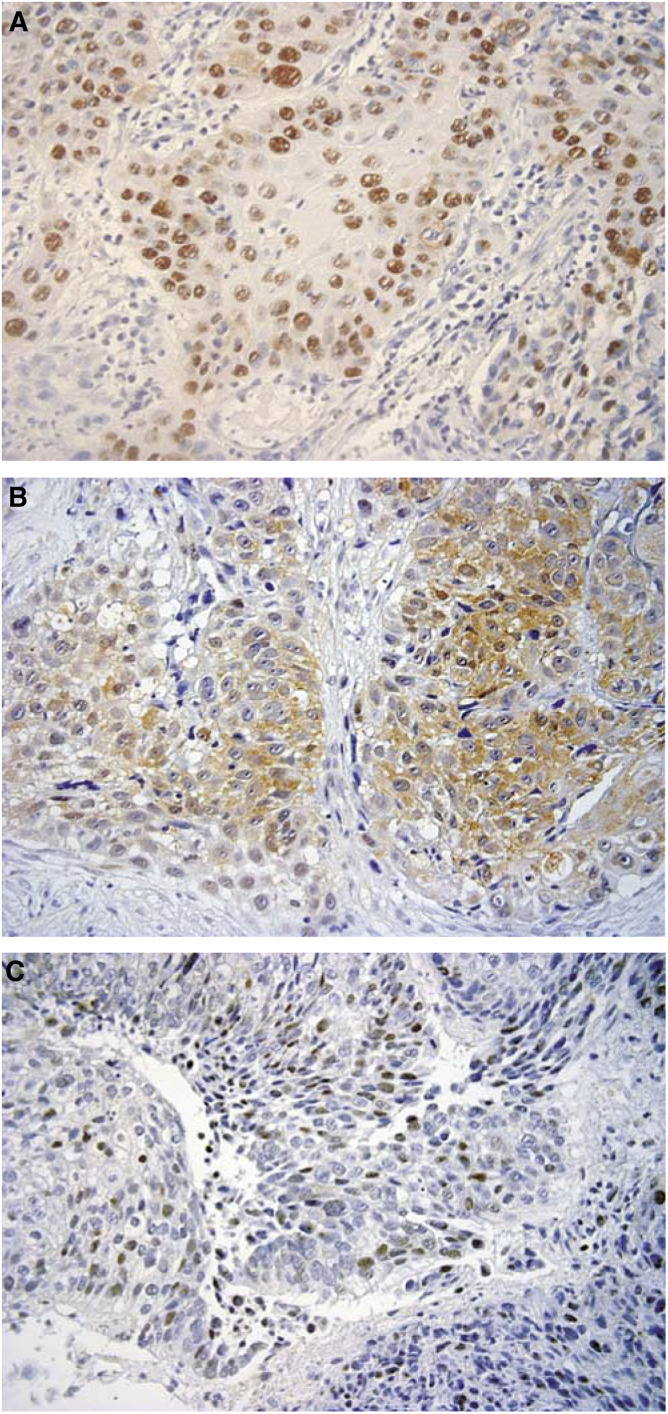
The expression of p53, p53R2, and p21 in oesophageal squamous cell carcinoma. The positive expressions of p53 and p21were found in the nuclei of cancer cells. The p53R2 expression was slightly detectable in the perinuclear and other cytoplasmic regions. **A**: p53(× 400); **B**: p53R2 (× 400); **C**: p21(× 400).

The p53 and p21 expressions were detected as nuclear staining; p53 had 43.5% positive expression and p21 had 54.8% positive expression. The p53R2 expression was slight detectable in perinuclear regions and distinct detectable in other cytoplasmic regions; p53R2 had 37.1% positive expression (Figure 1).

### Relationships between p53, p53R2, and p21 expressions and response to CRT

The percentage for clinical response of CR, PR, NC, and PD was 1.6% (one out of 62), 69.4% (43 out of 62), 27.4% (17 out of 62), and 1.6% (one out of 62), respectively. A total of 44 patients (71.0%) with CR or PR were judged as effective, whereas 18 patients (29.0%) with NC or PD were judged as not effective. The histological response rate of 36 patients was as follows: grade 1, 47.2% (17 out of 36 patients); grade 2, 30.6% (11 out of 36 patients); grade 3, 22.2% (eight out of 36 patients). In all, 19 patients (52.8%) with grade 2 or 3 were judged as effective, whereas 17 patients (47.2%) with grade 1 were judged as not effective. When comparing the relationship between the expression of p53 or p53R2 and the clinical response to CRT, CRT was effective in patients who had p53 (−) tumours (*P*=0.0001) or p53R2 (−) tumours (*P*=0.0013). However, no significant difference was found between p21 expression and clinical effect (*P*=0.70, data not shown) (Table 2). When the correlation of p53, p53R2, and p21 expression with histological effect was analysed, CRT was effective in p53 (−) tumours (*P*=0.041) or p53R2 (−) tumours (*P*=0.0018), whereas p21 expression did not influence histological effect (*P*=0.68, data not shown) (Table 3).

### Relationship between p53R2 expression and histological response to CRT according to p53 expression

When the relationship between p53R2 expression and the histological effect of CRT was analysed according to p53 expression, significant differences were found between the negative expression of p53R2 and the histological effect of CRT in p53 (−) tumours (*P*=0.0014). Particularly, all patients with grade 3 tumours had p53 (−) and p53R2 (−) tumours. On the other hand, p53R2 expression did not influence histological effect in p53 (+) tumours (*P*=0.15) (Table 4).

### Clinical outcomes according to p53 and p53R2 expression or CRT response

When comparing the relationship between the expression of p53 and p53R2, and clinical outcome in 62 patients, the 5-year survival rates of the patients with p53 (−) and p53 (+) tumours were 49.4 and 19.1%, respectively (*P*=0.0011), and p53R2 (−) and p53R2 (+) tumours were 43.2 and 23.0%, respectively (*P*=0.0057). According to the clinical response to CRT, the 5-year survival rate in patients with CR+PR and NC+PD tumours were 46.1 and 15.1%, respectively (*P*=0.001). According to multivariate analysis, although p53 and p53R2 expression was significant prognostic factors, clinical response to CRT was not selected as a prognostic factor (Table 5a).

**Figure 2:**
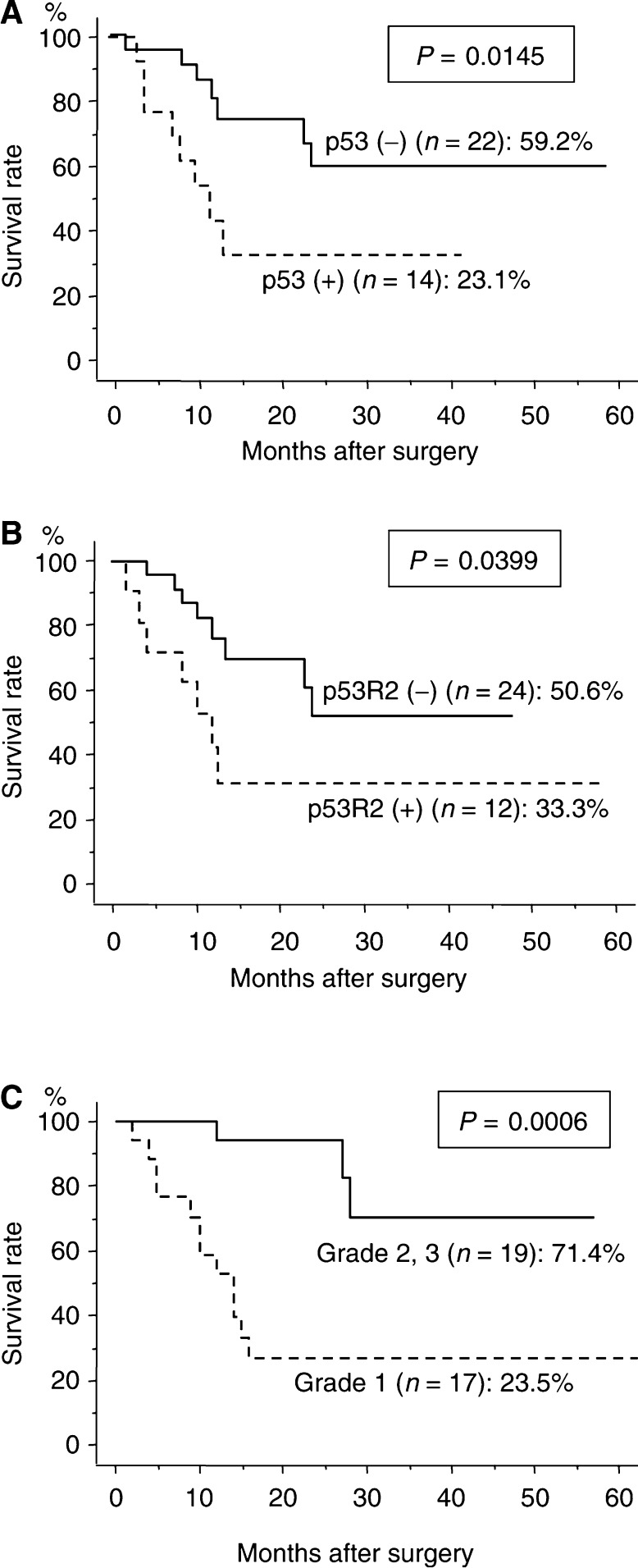
Cause-specific survival curves for ESCC patients treated by CRT and surgery (*n*=36), according to the expression of p53, p53R2, and pathological response. Survival curves were classified by p53 expression (**A**), p53R2 expression (**B**), and pathological response (**C**). The 5 year survival rates are indicated for each curve. *P*-values were calculated using logrank tests.

When analysing clinical outcome according to p53, p53R2, and pathological response to CRT in 36 patients who underwent surgery, the 5-year survival rates of the patients with p53 (−) and p53 (+) tumours were 59.2 and 23.1%, respectively (*P*=0.0145), p53R2 (−) and p53R2 (+) tumours were 50.6 and 33.3%, respectively (*P*=0.0399), and grade 2 or 3, and grade 1 tumours were 71.4 and 23.5%, respectively (*P*=0.0006) (Figure 2). Multivariate analysis revealed that only histological classification of response to CRT was significant prognostic factor (Table 5b).

**Figure 3:**
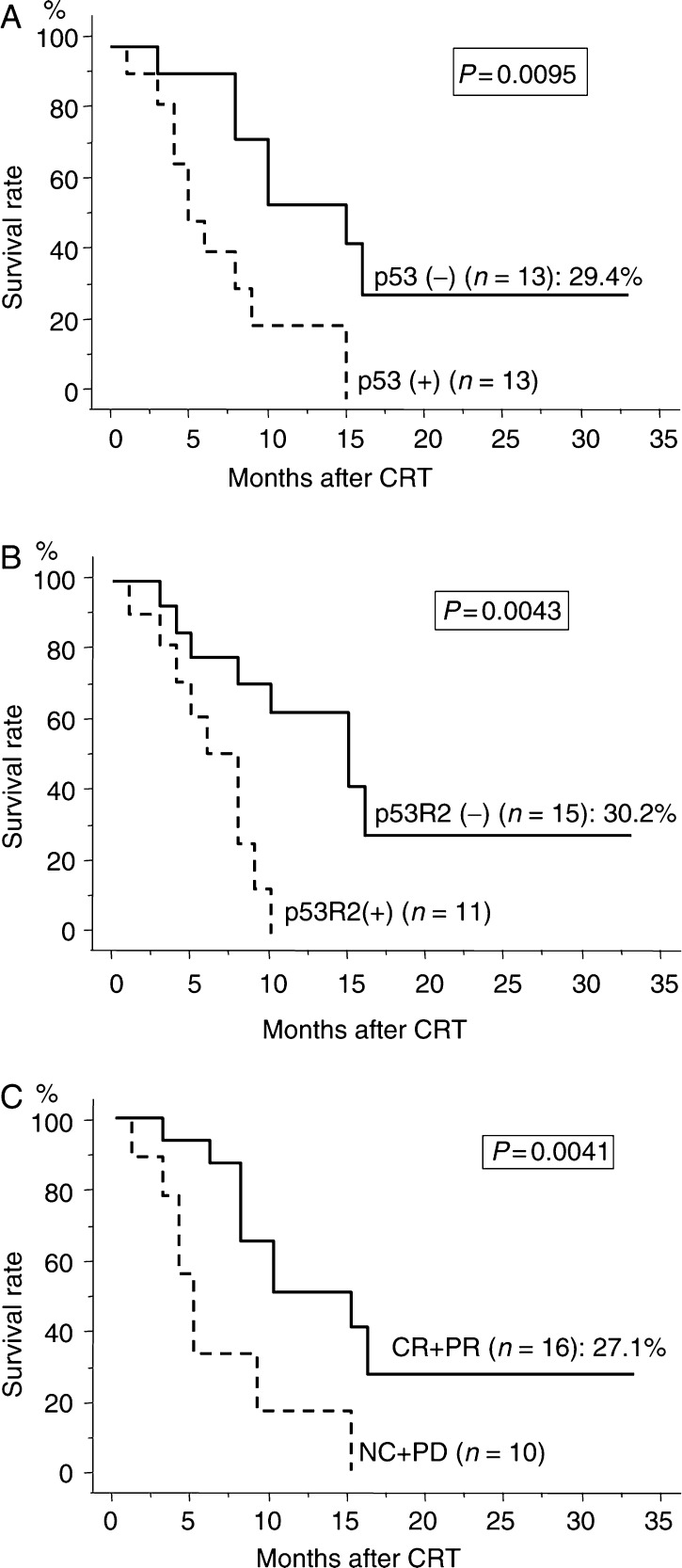
Cause-specific survival curves for ESCC patients treated by CRT alone (*n*=26), according to the expression of p53, p53R2, and clinical response. Survival curves were classified by p53 expression (**A**), p53R2 expression (**B**), and clinical response (**C**). The 3 year survival rates are indicated for each curve. *P*-values were calculated using logrank tests.

Additionally, when analysing clinical outcome according to p53, p53R2, and clinical response to CRT in 24 patients who did not undergo surgery, the 1- and 3-year survival rates of the patients with p53 (−) and p53 (+) tumours were 55.4, 29.4 and 21.0, 0%, respectively (*P*=0.0095), p53R2 (−) and p53R2 (+) tumours were 62.9, 30.2 and 0, 0%, respectively (*P*=0.0043), and CR+PR and NC+PD tumours were 50.8, 27.1 and 16.7, 0% respectively, (*P*=0.0041) (Figure 3).

## DISCUSSION

In the present study, we examined the expression of the proteins p53, p53R2, and p21 in ESCC to determine whether such expression was useful for predicting the response to CRT. This study showed a significant correlation between p53 (−) or p53R2 (−) expression and the clinical and histological effects of CRT. It was previously reported that p53 expression is a good marker for response to CRT in ESCC (Sarbia *et al*, 1998; Kishi *et al*, 2002). Although p53 may play a critical role in radiation-induced apoptosis, some patients with p53 (−) tumours do not respond well to CRT. A p53 (−) condition in immunohistochemical staining is associated with the wild-type p53 gene; however, some p53 (−) tumours with poor response to CRT may be associated with a complete loss of p53 protein due to a p53-null mutation, an acceleration of protein degradation (Hollstein *et al*, 1991), or the existence of another molecule that regulates radiation sensitivity in the p53 signalling pathway. Previously we reported that a combination of p53 (−) and p21 (+) expressions is useful for predicting the chemotherapeutic histological effect in ESCC (Nakashima *et al*, 2000). However, in the current study, we could not find a correlation between p21 (+) expression and the clinical and histological effects of CRT. This suggests that the mechanism of cell death in CRT is different from that in chemotherapy.

The expression of p53R2 is induced by wild-type p53 in response to various genotoxic stresses, including *γ*-irradiation, UV-irradiation, and exposure to anticancer drugs (Tanaka *et al*, 2000; Yamaguchi *et al*, 2001). DNA synthesis in cells arrested in G1 or G2 after DNA damage is mediated through the ribonucleotide reductase activity of p53R2. For cell survival, p53 activated by DNA damage then induces p53R2 expression to repair the damaged DNA. For cell death, either severe DNA damage or inactivation of the p53R2-dependent DNA synthesis pathway induces the apoptosis signalling pathway to eliminate the unrepaired cells (Tanaka *et al*, 2000). In the present study, p53R2 (−) expression was closely related to the clinical and histological effects of CRT. The histological effect was predictable in 17 of 24 patients on the basis of the p53R2 (−) expression results of the biopsy specimens. Furthermore, a positive response to CRT could be predicted in 13 of 14 patients (93%) on the basis of the combined results of p53 (−) and p53R2 (−) expression (Table 4). These results suggest that p53R2 has a DNA repair function downstream in p53. However, four of 10 patients (40%) with both p53 (+) and p53R2 (−) expression responded well to CRT. This result suggests that some tumours with a p53 mutation might be more susceptible to CRT than tumours with wild-type p53, because a lack of wild-type p53 could not induce arrest at G1 and thus reduced the time for DNA repair (Kastan *et al*, 1991; Vogelstein and Kinzler, 1992). Taken together, our results suggest that a good response to CRT for ESCC might be related to the efficiency of the DNA repair function in cancer cells. In the future, we hope to develop a positive response to CRT by inhibiting the function of DNA repair in ESCC.

Concerning the survival analysis, p53 (−) and p53R2 (−) were good prognostic factors in all patients of this study (*n*=62, study group), even if these patients were divided into two; in the patients treated with CRT followed by oesophagectomy (*n*=36, surgical group) and in the patients treated with only CRT (*n*=26, nonsurgical group). The clinical response to CRT was a prognostic factor in this study group. However, in surgical group, the clinical response to CRT was not selected as a prognostic factor although the pathological response was sole prognostic factor. With regard to discrepancy of prognostic factor between clinical and histological response to CRT, the tumours with clinical PR was included in various histological effects, grades 1, 2, and 3. In particular, 10 patients with clinical PR had grade 1 tumour by histological examination (Table 3). Further detailed analysis should be required in the discrepancy between clinical and histological response to CRT.

In conclusion, p53 (−) and p53R2 (−) expression in biopsy specimens of primary tumours is associated with a favourable effect of CRT for ESCC. Patients displaying these expressions may be good candidates for CRT. As immunohistochemical analysis of biopsy specimens for p53 and p53R2 expression is a simple and inexpensive test, these expressions should be evaluated before treatment.

## Figures and Tables

**Table 1:** Characteristics of patients

**Characteristics**	**No.**
Gender (male/female)	61/1
Age (years)	64 (48–77)
Tumour location	
Upper/middle/lower	15/34/13
Histological type	
Well/mode/poor	10/32/20
T	
T1/T2/T3/T4	2/3/35/22
N	
N0/N1	13/49
M	
M0/M1	48/14

**Table 2:** Correlation of p53 and p53R2 expression with clinical response to CRT

	**Clinical response to CRT (*n*=62)**					
	**CR**	**PR**	**NC**	**PD**	**Total**	***P***
p53						
(+)	0	12	14	1	27	0.0001
(−)	1	31	3	0	35	
p53R2						
(+)	0	12	10	1	23	0.013
(−)	1	31	7	0	39	

p53(+)/(−)=p53 positive/negative expression; p53R2(+)/(−)=p53R2 positive/negative expression; *P*-value was estimated as CR+PR *vs* NC+PD; CR=complete response; PR=partial response; NC=no change; PD=progressive disease.

**Table 3:** Correlation of p53, p53R2 expression, and clinical response with histological response to CRT

	**Histological response to CRT (*n*=36)**				
	**Grade 1**	**Grade 2**	**Grade 3**	**Total**	***P***
p53					
(+)	10	2	2	14	0.041
(−)	7	9	6	22	
					
p53R2					
(+)	10	2	0	12	0.0018
(−)	7	9	8	24	
					
Clinical response					
CR	0	0	0	0	0.0234
PR	10	10	8	28	
NC	7	1	0	8	
PD	0	0	0	0	

p53(+)/(−)=p53 positive/negative expression; p53R2(+)/(−)=p53R2 positive/negative expression; CR=complete response; PR=partial response; NC=no change; PD=progressive disease.

**Table 4:** Correlation of combination p53 and p53R2 expression with histological response to CRT

	**Histological response to CRT (*n*=36)**					
	**Grade 1**	**Grade 2**	**Grade 3**	**Total**		***P***
p53 (−) p53R2 (−)	1	7	6	14	]	0.0014
p53 (−) p53R2 (+)	6	2	0	8		
p53 (+) p53R2 (−)	6	2	2	10	]	0.15
p53 (+) p53R2 (+)	4	0	0	4		

p53(+)/(−)=p53 positive/negative expression; p53R2(+)/(−)=p53R2 positive/negative expression.

**Table 5:** Risk factors affecting the overall survival rate determined by univariate and multivariate analysis of p53, p53R2, and the clinical response (a) or pathological response (b) to CRT in 62 and 36 ESCC patients, respectively

	***P***		**Risk**	
	**Univariate**	**Multivariate**	**ratio**	**95% CI**
(a)				
p53 (−) *vs* p53 (+)	0.0011	0.0215	2.688	1.157–6.250
p53R2 (−) *vs* p53R2 (+)	0.0057	0.0185	2.469	1.164–5.235
CR+PR *vs* NC+PD	0.0009	0.2864	1.576	0.683–3.636
				
(b)				
p53 (−) *vs* p53 (+)	0.0145	0.1462	2.188	0.709–10.101
p53R2 (−) *vs* p53R2 (+)	0.0399	0.3059	1.515	0.566–6.098
Grade 2, 3 *vs* Grade 1	0.0006	0.0466	4.176	1.025–26.824

p53(+)/(−)=p53 positive/negative expression; p53R2(+)/(−)=p53R2 positive/negative expression; CRT=chemoradiation therapy; CR=complete response; PR=partial response; NC=no change; PD=progressive disease.
